# Highly Coupled Seven-Core Fiber for Ratiometric Anti-Phase Sensing

**DOI:** 10.3390/s23167241

**Published:** 2023-08-18

**Authors:** Natanael Cuando-Espitia, Andrés Camarillo-Avilés, Daniel A. May-Arrioja, Iván Hernández-Romano, Miguel Torres-Cisneros

**Affiliations:** 1 CONACyT-Electronics Department, University of Guanajuato, Carr. Salamanca-Valle de Santiago Km 3.5 + 1.8, Salamanca 36885, Mexico; hromano@ugto.mx; 2 Centro de Investigaciones en Óptica, Prol. Constitución 607, Fracc. Reserva Loma Bonita, Aguascalientes 20200, Mexico; a.camarillo.aviles@cio.mx (A.C.-A.); darrioja@cio.mx (D.A.M.-A.); 3 Applied Physics Group, DICIS, University of Guanajuato, Carr. Salamanca-Valle de Santiago Km 3.5 + 1.8, Salamanca 36885, Mexico; torres.cisneros@ugto.mx

**Keywords:** fiber optic sensors, multicore fiber, thermal sensors

## Abstract

A ratiometric fiber optic temperature sensor based on a highly coupled seven-core fiber (SCF) is proposed and experimentally demonstrated. A theoretical analysis of the SCF’s sinusoidal spectral response in transmission configuration is presented. The proposed sensor comprises two SCF devices exhibiting anti-phase transmission spectra. Simple fabrication of the devices is shown by just splicing a segment of a 2 cm long SCF between two single-mode fibers (SMFs). The sensor proved to be robust against light source fluctuations, as a standard deviation of 0.2% was registered in the ratiometric measurements when the light source varied by 12%. Its low-cost detection system (two photodetectors) and the range of temperature detection (25 °C to 400 °C) make it a very attractive and promising device for real industrial applications.

## 1. Introduction

Ratiometric sensors have been widely used in electrochemical analysis [1,2,3,4,5,6] and fluorescence-based sensing [7,8,9,10,11]. For example, ratiometric sensors have been developed to detect and measure DNA [12,13], glucose [14,15], and metal ions [16,17,18]. Among the advantages of ratiometric sensing schemes, one may mention the robustness over fluctuations, high accuracy, and great reproducibility. The basic idea of ratiometric sensing is the readout of two characteristic signals from the target measurand. When properly selected, one signal may act as a reference, making the ratio between them source-independent and providing the sensor with immunity against spurious signal fluctuations. Moreover, the characteristic signals may exhibit intensity changes in opposite directions, allowing an enhanced sensitivity. This improved sensitivity is particularly beneficial for sensing conditions with a low signal-to-noise ratio.

On the other hand, and due to their materials and fabrication, fiber optic sensors share advantages such as compact size, light weight, immunity to electromagnetic interference, and chemical inertness. Therefore, fiber optic sensors have been developed to operate in harsh environments [19,20] and have shown reliability in measuring optical and mechanical variables such as refractive index [21,22], curvature [23,24], surface tension [25,26], and temperature [27,28]. Ratiometric sensing has been implemented in optical fibers to measure oxygen concentration [29,30], pH [31,32,33], and antibiotics [34]. In general, ratiometric fiber sensors use engineered fluorophores attached to optical fibers, generating a convenient optical probe for in-site measurement. In the experimental implementation, the fiber is used to launch the excitation irradiation as well as to collect the fluorescence emission. Then, the spectral emission is analyzed, and the ratio between two specific wavelengths is measured.

Recently, we reported a ratiometric temperature sensor based on two sections of highly coupled multicore fibers (MCFs) [35]. The designed sensor reported in [35] relied on the spectral features of the MCFs, which means that no fluorophore was required. Briefly, the sensor’s spectral response is a function of the MCF geometry, MCF fiber lengths, and thermo-optic coefficient of the sensor materials [36]. The transmission spectrum of the proposed sensor exhibited a series of peaks within the near-infrared region. Based on numerical simulations, we tuned the MCF lengths in order to observe two wavelength peaks with opposite behavior in intensity when the MCFs were subjected to temperature variations. The MCF-based ratiometric sensor was simple to construct while exhibiting an enhanced sensitivity compared to non-ratiometric measurements. The main drawback of this approach was the use of expensive equipment, such as a supercontinuum (SC) light source and an optical spectrum analyzer (OSA) in the detection stage.

Applications such as point-of-care biosensing and industrial 4.0 sensors benefit from robust, portable, and inexpensive devices. Although optical fiber and fiber devices are inexpensive, the combined costs of an SC light source and an OSA exceed USD 45k, clearly preventing further applications. With that in mind, and in contrast to the approach reported in [35], we have designed, simulated, constructed, and tested an MCF-based ratiometric temperature sensor implemented with a commercially available and inexpensive light source and optical detectors. By connecting an NIR laser diode to two MCF sections in parallel and carefully selecting the spectral response of individual MCF sections, we obtained ratiometric operation from the voltage registered in two optical detectors. In this report, we combine the advantages of ratiometric sensing and the convenient implementation of fiber optic sensors. We believe these results represent a significant advance towards a more extensive development of industrial and MCF-based sensors.

## 2. Principle of Operation

According to coupled-mode theory, the transmission spectrum of a seven-core highly coupled fiber (SCF) has a sinusoidal form with spatial frequency *β* and intrinsic phase *α* [22]. Then, one can model the normalized transmission response *I* of an SMF-SCF-SMF device as the following general expression:

(1) $$I\left(\lambda \right)=\frac{1}{2}\left\{1+cos\left(\beta \lambda +\alpha \right)\right\}.$$

**Figure 1:**
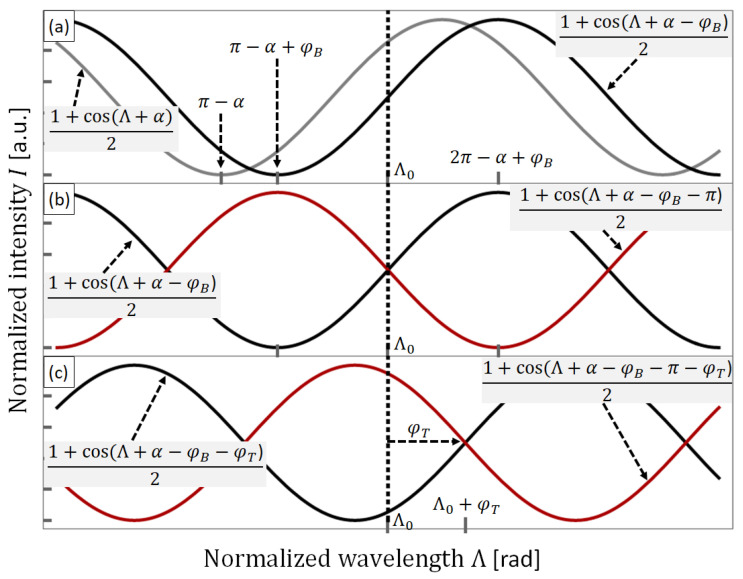
(**a**) Generalized spectral response of an SMF-SCF-SMF device (grey curve) and after a curvature-induced phase shift (black curve). (**b**) Spectral response of the previous curved device (black curve) and the spectral response of a similar device shifted π radians (red curve). (**c**) Spectral response of the two anti-phase devices after a temperature-induced phase shift. See the text for more details.

In Equation (1), *λ* indicates wavelength, and the performed normalization restricts intensity to runs from 0 to 1. In general, *β* and *α* depend on the geometry, refractive indices, and fiber length. The inverse of spatial frequency is sometimes referred to as the free spectral range (FSR) and is important in optical sensors as it determines the operation range of the device. In other words, the FSR of an optical sensor should be larger than the maximum spectral shift to avoid ambiguity in the measurements. Thus, the spatial frequency, temperature, and Δ*T_max_* of an SCF-based sensor should meet the relationship:

(2) $$\frac{2\pi }{\beta }>{\Delta T}_{max}\frac{\partial \varphi }{\partial T}$$

where ∂*φ*/∂*T* indicates the phase shift with respect to temperature changes. Expressing Equation (1) in terms of a normalized wavelength Λ = *βλ* allows us to study the induced phase shift as a function of curvature and temperature. Figure 1a shows the generalized response of an SMF-SCF-SMF device of spatial frequency *β* and intrinsic phase shift *α* as a solid grey line. The normalized intensity of this device exhibits a minimum when the normalized wavelength is equal to π + α and a maximum when Λ is equal to 2π + α as depicted in Figure 1a. We have indicated the operation wavelength in Figure 1 as a vertical dotted line and labeled it Λ_0_. On the other hand, it has been demonstrated that bending SMF-MCF-SMF devices translates into a high-sensitivity spectral shift [37,38,39,40]. Therefore, the spectral response of the proposed device can be set to intersect the operation wavelength at half the normalized intensity, provided a bending-based shift *φ_B_*. The operation point at half the intensity is selected as the sinusoidal response is highly linear around this point. Figure 1a depicts as a solid black curve, the simulated response of the device after a shift of *φ_B_*. Moreover, we can obtain an anti-phase version of the previous device using the same SCF lengths and inducing a curvature to shift the spectral response by *φ_B_* + π radians. Figure 1b shows the response of the first device as a solid black curve and the response of the anti-phase device as a solid red curve. Notice that both curves intersect the operation wavelength at half the normalized intensity.

Although the temperature may alter the spatial frequency via refractive index variations, we have shown that the main contribution of temperature in an SMF-SCF-SMF device is a phase shift in the spectral sinusoidal response [22]. Then, in terms of temperature and bending disturbances, the normalized intensity of an SMF-SCF-SMF device can be modeled as follows:

(3) $$I\left(\Lambda \right)=\frac{1}{2}\left\{1+cos\left(\Lambda +\alpha -\varphi_{B}-\varphi_{T}\right)\right\}$$

Figure 1c shows the simulated response of the same devices in Figure 1b with an additional phase shift of *φ_T_* caused by increasing the temperature of both SMF-SCF-SMF devices. As shown in Figure 1c, the vertical line intersects both curves at different normalized intensity values. Thus, the temperature change may be tracked by recording the values of both devices at the operation wavelength. Moreover, by performing a ratiometric measurement of the recorded values, the measurement can be independent of the actual optical power supplied by the light source. We can further analyze the temperature response of the ratiometric measurements at Λ_0_ as follows:

(4) $$\frac{I_{2}\left(\Lambda_{0}\right)}{I_{1}\left(\Lambda_{0}\right)}=\frac{1+cos\left(\Lambda_{0}+\alpha -\varphi_{B}-\varphi_{T}-\pi \right)}{1+cos\left(\Lambda_{0}+\alpha -\varphi_{B}-\varphi_{T}\right)}=\frac{1-\sin\varphi_{T}}{1+\sin\varphi_{T}}$$

**Figure 2:**
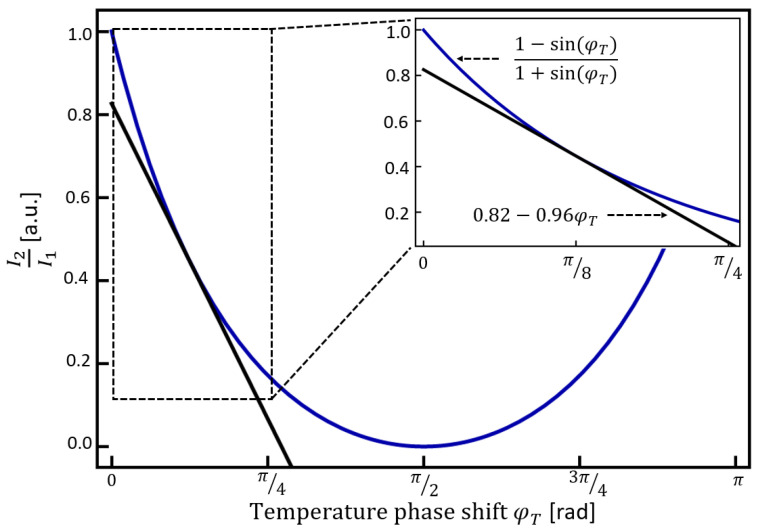
Ratiometric measurement (see Equation (4)) as a function of temperature-induced phase shift.

In Equation (4), the fact that the operation point was conveniently set to cos(Λ_0_ + α − *φ_B_*) = 0 was used to simplify the expression of the ratiometric response. Figure 2 shows the ratiometric response as a function of temperature-induced phase shift as expressed in Equation (4).

Although Figure 2 shows a clear non-linear behavior, the inset of Figure 2 shows the corresponding response from 0 to π/4, in which a moderate degree of linearity is appreciable. In order to show the linear tendency of the ratiometric response between 0 and π/4, we have calculated the first-order Taylor series around π/8 and plotted it in Figure 2 as a solid black curve. Thus, the linear approximation of Equation (4) around π/8 was found as 0.82 − 0.96*φ_T_*, as indicated in the inset of Figure 2. According to this analysis, a ratiometric anti-phase sensor based on SCF can exhibit a good linear response for temperature increments that translate to phase shifts of π/4 or less.

## 3. Sensor Construction and Experimental Setup

**Figure 3:**
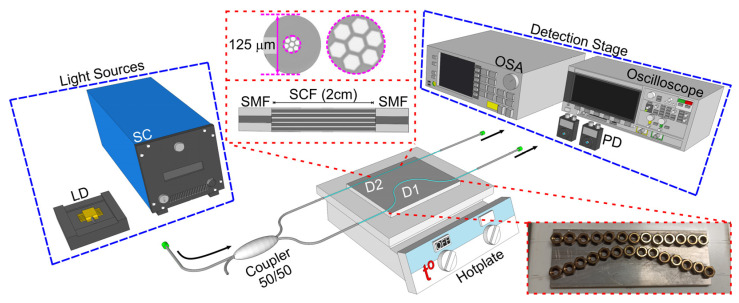
Schematic representation of the experimental setup. LD: laser diode, SC: supercontinuum light source, OSA: optical spectrum analyzer, PD: photodiode, D1, D2: SMF-SCF-SMF devices. The top middle inset depicts the transversal section of the SCF used in this work. The bottom right inset shows a picture of the aluminum plate inscribed with the two channels. Several stainless-steel nuts were placed over the channels to keep the fiber in place.

Based on the results of the previous section, an SCF exhibits several advantages, such as interferometric-like behavior, tunability of its spectral response, and a temperature-induced spectral shift. Thus, we constructed two SMF-SCF-SMF devices with an SCF length of 2 cm. An SCF cross-section photograph is shown in Figure 3. The SCF structure presents a hexagonal central core and six external hexagonal cores, which are symmetrically positioned with respect to the faces of the central core. The refractive indices of the cores and cladding are 1.450 and 1.444, respectively. The center-to-center separation between the cores is 11 μm, and the size of the core measured between two hexagonal edges is 9 μm which provides an edge separation of about 2 μm. Additionally, the SCF cladding has a conventional 125 μm diameter. The Microstructured Fibers and Devices Group at CREOL-UCF provided the SCF to construct these devices. The SCF section was first spliced to an SMF using a fusion splicer (70S, Fujikura, Tokyo, Japan), where a multimode splicer mode was employed. This SMF-SCF union was displaced 2 cm through a micrometric mount with 0.01 mm resolution and 50 mm maximum displacement. Then, it was cleaved and finally spliced to another SMF. Care was taken to remove all polymer cladding in both SMF-SCF-SMF structures, where the final length exposed to temperature changes was about ~16 cm. To induce a bending-based spectral shift, we engraved a curved channel on a 14 cm × 6 cm × 0.5 cm aluminum plate using a fiber-based laser engraver (50W XT Laser, Shandong, China). The depth of the channel was 200 µm, and the curve shape was determined by gently adjusting the curvature of the SMF-SCF-SMF device until the desired spectral shift was obtained. As we will detail in the following section, the spectral response of one of the experimental devices showed the expected anti-phase behavior at 1550 nm without bending, which led us to engrave a curve and a straight channel on the same aluminum plate. The curvature radius of the curved channel was approximately 5.43 m^−1^ (18.40 cm).

As schematically shown in Figure 3, the aluminum plate was placed over a hotplate (Cimarec, SP88850100, ThermoScientific Waltham, MA, U.S.A.), and the SMF-SCF-SMF devices were allocated to the scribed channels, making sure to accommodate the SCF segments at the center of the channels. Additional stainless-steel nuts were placed over the channels to maintain the fibers within the channels and minimize contact with ambient air. As depicted in Figure 3, two light sources were used in this series of experiments; a supercontinuum source (SC, SC500, FYLA, Valencia, Spain) was used for the spectral characterization, while a laser diode (LD) at 1550 nm (LDM 1550-DB-1-FA) was used in the single-wavelength implementation. Similarly, an optical spectrum analyzer (OSA, MS9740A, Anritsu, Atsugi, Kanagawa, Japan), two photodetectors (PDs, PDA20C, Thorlabs, Newton, NJ, U.S.A.), and a 100 MHz oscilloscope (DSOX2012A, Keysight, Santa Rosa, CA, U.S.A.) were used in the detection stage for the spectral characterization and single-wavelength implementation, respectively. As shown in Figure 3, a 50/50 fiber coupler was used to split the input light at both device entries. For the single-wavelength implementation, the light source was controlled using an LD current controller (LDC220C, Thorlabs, Newton, NJ, U.S.A.) with a 100 µA resolution, an accuracy of about ±2.0 mA, and ±2.0 A current operation control range. For both heat characterizations, the hotplate varied its temperature from 25 °C to 400 °C in steps of 25 °C. This controlled temperature change produced an anti-phase redshift in both devices that will be shown and described in the next section.

## 4. Results

### 4.1. Spectral Characterization

**Figure 4:**
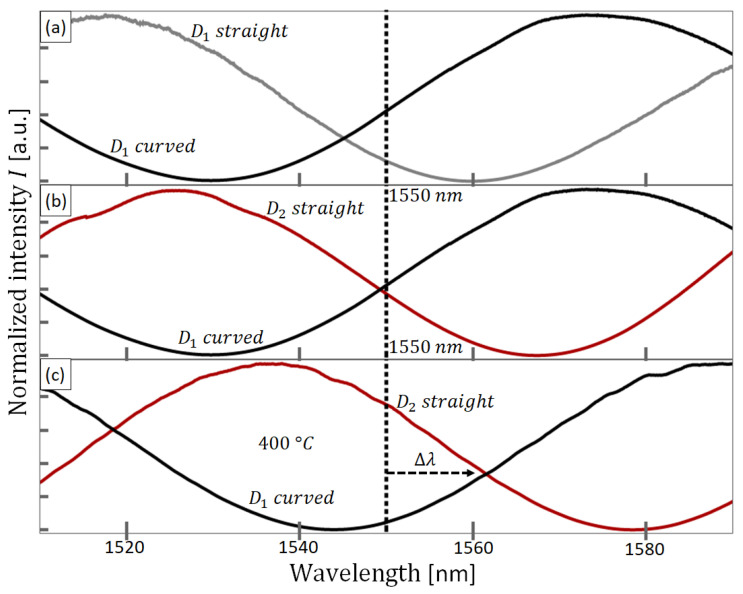
Experimental results of spectral characterization. (**a**) Registered spectrum of D1 device as fabricated (grey curve) and after being placed in the inscribed curved channel (black curve). (**b**) Experimental spectrum of curved D1 device (black curve) and spectrum of as-fabricated (straight) D2 at 25 °C (red curve). (**c**) Experimental registered spectra of both devices heated at 400 °C. Black curve in (**c**) corresponds to curved D1 spectrum while the red curve in (**c**) corresponds to straight D2 spectrum.

The normalized spectrum recorded from the first SMF-SCF-SMF device (D1) is shown in Figure 4a as a solid grey curve. The operation wavelength, 1550 nm, is indicated in Figure 4 as a vertical broken line. As expected, a sinusoidal response is observed. The measured spatial frequency was 78.5 rad/µm (FSR~80 nm), close to other reports using similar devices [36,41,42]. As previously mentioned, the D1 device was carefully curved while registering the corresponding spectra until the measured spectrum intersected the operation wavelength at around 0.5 of the normalized intensity. Once the desired intersection was obtained, the fiber device was temporally fixed with tape, and a picture of the curved device was taken. The picture was used as a template to inscribe a curved channel on an aluminum plate, and the fiber device was set into the scribed channel. The experimental spectrum of the curved D1 device is shown as a solid black curve in Figure 4a. Notice that the intersection with the operation wavelength occurs close to half the normalized intensity.

Then, we obtained the transmission spectrum of device D2. The corresponding spectrum of D2 is shown in Figure 4b as a solid red line. For this particular device, the target spectral response was found without the need for additional bending. Thus, a straight channel was engraved for D2 on the same aluminum plate. In addition to the spectrum of D2, Figure 4b shows the corresponding spectrum of D1 within the curved channel, showing the anti-phase operation point at ambient temperature (25 °C). To investigate the thermal response of the devices, we registered spectra of both devices at different temperatures from ambient temperature to 400 °C in steps of 25 °C. Figure 4c shows the measured spectra at 400 °C. Figure 4c shows a wavelength shift of 12 nm, which yields a spectral sensitivity of ∂λ/∂T = 0.032 nm/°C. This spectral sensitivity is also similar to other reports [28,42] and corresponds to 0.9 radians compared to the measured spatial frequency.

In contrast to the operation point at ambient temperature (Figure 4b), in which the normalized intensities of both devices are very similar, at 400 °C the intensity of D1 has decreased while the intensity of D2 has increased (Figure 4c). This behavior results from the opposite slopes seen in the operation point at ambient temperature and the fact that temperature increments shift both spectra to longer wavelengths. Based on the results shown in Figure 4, we can expect that, when using a single-wavelength light source at the designed operation wavelength and optical detectors in the detection stage, the ratiometric computation D2/D1 provides robust and source-independent temperature measurements.

### 4.2. Single-Wavelength Implementation

**Figure 5:**
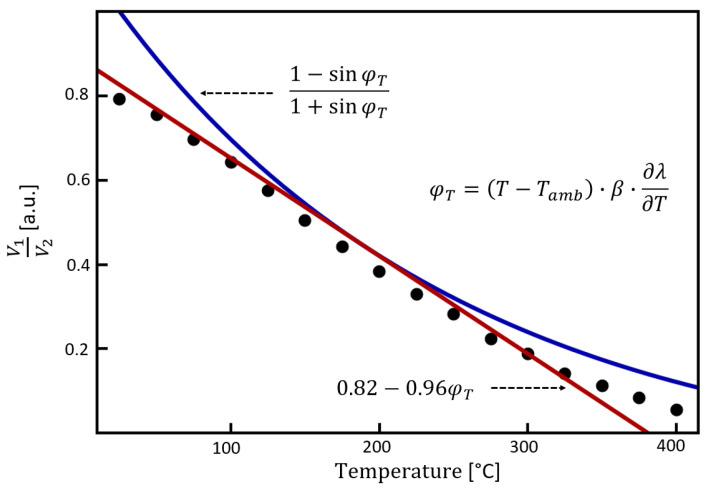
Experimental ratiometric measurements V1/V2 as function of temperature (black circles). The blue curve corresponds to the theoretical response using Equation (4), while the red curve represents the linear approximation described in Section 2 (see Figure 2).

Once the spectral characterization was completed, we implemented the single-wavelength experiment, using an LD operating at 1550 nm as a light source and two photodetectors (PD1 and PD2) in the detection stage. As shown in Figure 3, a standard oscilloscope (DSOX2012A, Keysight, CA, U.S.A.) was used for monitoring the voltage of the PD1 (V1) connected to D1 and PD2 (V2) connected to D2, both using fiber optic connectors. The LD output was initially fixed for the ratiometric measurements to emit 3.06 mW of optical power. Subsequently, the hotplate temperature was varied in steps of 25 °C with a maximum temperature of 400 °C. To ensure a homogeneous temperature in the hotplate and both SCF devices, we registered the corresponding ratiometric measurement after ten minutes of setting the target temperature in the hotplate. Figure 5 summarizes the results of the single-wavelength implementation proposed here. The experimental data are depicted in Figure 5 as solid black circles, and the theoretical curve (Equation (4)) is shown as a solid blue line. In addition, the linear approximation of Equation (4) is shown in Figure 5 as a solid red curve. The theoretical curves show good agreement with the experimental data as R^2^ calculated using Equation (4) was 89% and 97% using the linear approximation of Equation (4). The observed deviations between data and theoretical curves may be related to device imperfections, such as dissimilarities in spatial frequency between fiber devices and intrinsic losses from fiber splices. In order to obtain *φ_T_*, we have used the experimental data collected in Section 4.1 and calculated the temperature-induced phase shift as ΔT∙∂λ/∂T∙*β*.

The results shown in Figure 5 demonstrate that fiber optic sensors based on MCFs may lead to robust, versatile, and inexpensive industrial sensors. Notice that we have selected V2 as the denominator to generate a linear decrease as the temperature increases. In other words, a temperature increment induces a redshift in both SCF devices, which produces a V1 decrement and a V2 increment. However, if the temperature is fixed and any power increment occurs in the light source, the registered voltage in both PDs would increase accordingly. Therefore, the ratiometric factor is independent of the source power fluctuations since any fluctuation will affect both fiber devices proportionally. The following section will describe a stability test performed on the proposed anti-phase sensor by inducing sinusoidal variations in the LD optical power.

### 4.3. Stability Tests

**Figure 6:**
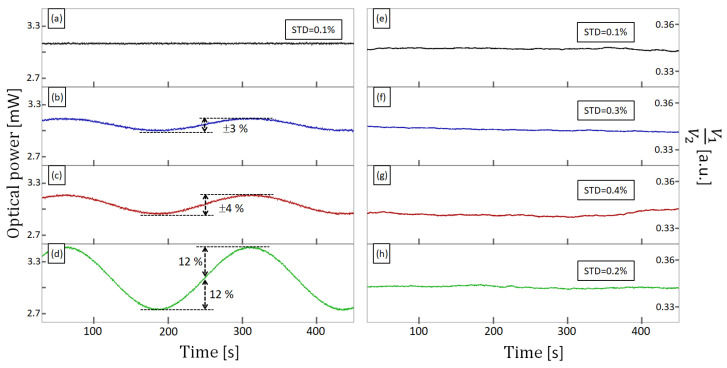
Stability test of the proposed sensor. (**a**–**d**) Optical power of the LD as a function of time. The optical power was modulated using a sinusoidal signal of 4 mHz and up to ±12% variation. (**e**–**h**) Corresponding ratiometric measurements. (**e**) corresponds to ratiometric measurement when the optical power of LD was varied, as shown in (**a**). Similarly, (**f**) corresponds to (**b**); (**g**) to (**c**); and (**h**) to (**d**).

To further explore the stability capabilities of the proposed sensor, we induced a sinusoidal variation in the optical power of the light source. With a fixed temperature of 200 °C at the hotplate, we initially set the pump current of the LD to 21 mA in the LD current driver. This current corresponds to an optical power of 3.1 mW at the LD output and translates to voltage readings of V1 and V2 of 0.33 V and 0.97 V, respectively. The ratiometric measurement of this particular case is 0.34, as V2 is about three times higher than V1. Figure 6a shows the registered optical power of the light source for 450 s when the current in the LD was fixed at 21 mA. Similarly, Figure 6e presents the ratiometric measurement for the previously described case for 450 s. As seen in Figure 6e, the ratiometric measurement shows slight deviations from the expected value of 0.34 (standard deviation = 0.1%). Then, we fed the LD current driver with a sinusoidal signal of 4 mHz, allowing the optical power to run from 2.97 mW up to 3.15 mW, as shown in Figure 6b. This variation corresponds to ±3% of the optical power. Figure 6f shows the corresponding ratiometric measurement as a function of time when the optical source operates at 3.1 mW ± 3%. As shown in Figure 6f, the variation in optical power does not translate to a sinusoidal variation in the ratiometric measurement. The standard deviation of the data presented in Figure 6f corresponds to 0.3%.

Maintaining the mean optical power fixed at 3.1 mW and the frequency at 4 mHz, we also tested our sensor for optical power variations of ±4% and ±12%, as shown in Figure 6c and Figure 6d, respectively. The corresponding ratiometric measurements for ±4% and ± 12% optical power variation are presented in Figure 6g and Figure 6h, respectively. As in the previous experiment, no sinusoidal behavior can be seen in the curves of Figure 6g,h. The standard deviation of the data shown in Figure 6g is equivalent to 0.4%. Figure 6h shows remarkable stability (standard deviation = 0.2%) despite the light source being varied by 12%. The mechanism of this stability is related to the proportion between the voltage readings V1 and V2. Although the sinusoidal signal of the light source effectively modulates the voltage readings, both voltages increase and decrease simultaneously and proportionally. Therefore, the ratiometric measurement removes the variations related to the light source. The results shown in Figure 6 are important as they indicate that inexpensive light sources (typically with stabilities of less than ±10%) can be used to implement the sensing strategy proposed here.

## 5. Discussion and Conclusions

The results shown in previous sections demonstrate that a robust, highly linear, and easy-to-implement temperature sensor can be constructed based on short sections of highly coupled SCF by registering the quotient from two voltage signals. A key advantage of SCF-based sensors is their spectral tuning capabilities. In this exploration, we used that advantage to tune the linear response of two anti-phase SCF devices at a convenient operation wavelength to obtain enhanced sensitivity and good linearity performance.

We showed in Section 2 that a good linear response could be found for a temperature-induced phase shift of about π/4 (~0.78 radians). In Section 3, we confirmed that good linearity is experimentally observed even for slightly higher phase shift values than π/4, as the maximum shift observed of 12 nm corresponds to a phase shift of 0.9 radians. The slope of the linear approximation of the experimental data was 0.002 ratiometric units (R.U.) per centigrade. Notice that the slope of the experimental data shown in Figure 5 defines the sensitivity of the proposed sensor as ratiometric units per centigrade (R.U.°C^−1^), where ratiometric units result from the ratiometric measurement V1/V2. The experimental sensitivity value of 0.002 [R.U.°C^−1^] means that in each 100 °C step, the ratio between signals decreases by 20%, independently of the actual voltage readings. Indeed, temperature range and sensitivity are set once the fiber device is fabricated. However, designing a similar device for a predefined target temperature range or sensitivity is relatively easy. For instance, a sensor operating at twice the temperature range can be readily constructed by doubling the FSR and using half the SCF length (1 cm). Similarly, a more sensitive sensor can be designed using twice the SCF length (4 cm) to increase the slope from 20% to 40% in each temperature step of 100 °C.

Evidently, one disadvantage of this proposed sensor is that the sensitivity and temperature range are inversely proportional in this approach. Therefore, designing a sensor for a larger temperature range with high sensitivity can be difficult. Nevertheless, many high-temperature and biological applications such as engine tests, reaction chambers, metallurgical processes, cell culture monitoring, and biomicrofluidics would greatly benefit from an easy-to-implement, versatile, non-electrical, small, chemically inert, and biocompatible sensor that is robust over source fluctuations such as the anti-phase ratiometric fiber temperature sensor presented here.

A comparison of our proposed setup with similar previous works reported in [27,28,36,42,43] is shown in Table 1. In general, previous works rely on tracking the spectral shift of the engineered fiber device, and few efforts have been reported on high-temperature sensing (above 400 °C). To the best of our knowledge, we are the first research group to design and construct a ratiometric anti-phase fiber sensor for temperature sensing. Moreover, in this report, we propose and show a single-wavelength characterization system that allows robustness against power source fluctuations and avoids using expensive equipment.

Moreover, the promising results shown here indicate that further explorations on this sensing approach can be attractive. For example, due to the reduced size of fiber optics, this sensing approach should exhibit a rapid response in following the temperature of its surroundings, even in sub-zero temperatures. The implementation can also be revised to reduce the cost of the system, improve resolution, or emphasize automatization by removing LD current/temperature drivers, using balanced photodetectors in the detection stage, or replacing the oscilloscope with a microcontroller board. Finally, we believe this versatile and inexpensive approach may help to design and construct more robust and attractive industrial fiber optic sensors.

In summary, we have proposed and demonstrated a fiber optic temperature sensor based on an SCF that works in a ratiometric scheme. The inexpensive detection system of the device as well as its high sensitivity and temperature range (25 °C to 400 °C) make this sensor a good candidate for industrial applications.

## Figures and Tables

**Table 1:** Comparison of the ratiometric anti-phase sensor with recently reported SCF fiber temperature sensors [30,31,32,33].

Structure	Sensing Mechanism	Sensitivity		Temperature Range	Reference
		Spectral	R.U.		
FBGs inscribed in each SCF core	Sensing temperature through different curvatures	9.97 pm/°C	NA	30–70 °C	[27]
Two SMF-SCF-SMF devices	Temperature-induced spectral shift	321 pm/°C	NA	26–150 °C	[28]
SMF-SCF -SMF	Temperature-induced spectral shift	29 pm/°C	NA	22–134° C	[36]
SMF-SCF-SMF	Temperature-induced spectral shift	100–300 °C: ~28.7 pm/°C. 300–1000 °C: ~51.7 pm/°C	NA	100–1000 °C	[42]
SMF-SCF-SMF	Temperature-induced spectral shift	43 pm/°C	NA	25–1000 °C	[43]
Two SMF-SCF-SMF devices	Ratiometric temperature sensing	32 pm/°C	−0.0021 R.U./°C	25–400 °C	This work

## Data Availability

Data are contained within the article and more details are available on request from the corresponding author.

## References

[1] Wei J., Liu C., Wu T., Zeng W., Hu B., Zhou S., Wu L. (2022). A review of current status of ratiometric molecularly imprinted electrochemical sensors: From design to applications. Anal. Chim. Acta.

[2] Zhu C., Wang X., Yang Y., Chen L., Yu D. (2022). Research progress on ratiometric electrochemical sensing of mycotoxins. J. Electroanal. Chem..

[3] Yang T., Yu R., Yan Y., Zeng H., Luo S., Liu N., Morrin A., Luo X., Li W. (2018). A review of ratiometric electrochemical sensors: From design schemes to future prospects. Sens. Actuators B Chem..

[4] Jin H., Gui R., Yu J., Lv W., Wang Z. (2017). Fabrication strategies, sensing modes and analytical applications of ratiometric electrochemical biosensors. Biosens. Bioelectron..

[5] Cheng H., Wang X., Wei H. (2015). Ratiometric electrochemical sensor for effective and reliable detection of ascorbic acid in living brains. Anal. Chem..

[6] Yang J., Hu Y., Li Y. (2019). Molecularly imprinted polymer-decorated signal on-off ratiometric electrochemical sensor for selective and robust dopamine detection. Biosens. Bioelectron..

[7] Hou J., Jia P., Yang K., Bu T., Zhao S., Li L., Wang L. (2022). Fluorescence and Colorimetric Dual-Mode Ratiometric Sensor Based on Zr–Tetraphenylporphyrin Tetrasulfonic Acid Hydrate Metal–Organic Frameworks for Visual Detection of Copper Ions. ACS Appl. Mater. Interfaces.

[8] Gan Z., Zhang T., An X., Tan Q., Zhen S., Hu X. (2022). A novel fluorescence-scattering ratiometric sensor based on Fe-NC nanozyme with robust oxidase-like activity. Sens. Actuators B Chem..

[9] HeeáLee M., SeungáKim J. (2015). Small molecule-based ratiometric fluorescence probes for cations, anions, and biomolecules. Chem. Soc. Rev..

[10] Park S.H., Kwon N., Lee J.H., Yoon J., Shin I. (2020). Synthetic ratiometric fluorescent probes for detection of ions. Chem. Soc. Rev..

[11] Chen Y., Zhu C., Yang Z., Chen J., He Y., Jiao Y., He W., Qiu L., Cen J., Guo Z. (2013). A ratiometric fluorescent probe for rapid detection of hydrogen sulfide in mitochondria. Angew. Chem..

[12] Xie F.T., Zhao X.L., Chi K.N., Yang T., Hu R., Yang Y.H. (2020). Fe-MOFs as signal probes coupling with DNA tetrahedral nanostructures for construction of ratiometric electrochemical aptasensor. Anal. Chim. Acta.

[13] Gao F., Du L., Zhang Y., Tang D., Du Y. (2015). Molecular beacon mediated circular strand displacement strategy for constructing a ratiometric electrochemical deoxyribonucleic acid sensor. Anal. Chim. Acta.

[14] Wang L., Xu M., Xie Y., Qian C., Ma W., Wang L., Song Y. (2019). Ratiometric electrochemical glucose sensor based on electroactive Schiff base polymers. Sens. Actuators B Chem..

[15] Gong C., Shen Y., Song Y., Wang L. (2017). On-off ratiometric electrochemical biosensor for accurate detection of glucose. Electrochim. Acta.

[16] Yu Y., Yu C., Yin T., Ou S., Sun X., Wen X., Zhang L., Tang D., Yin X. (2017). Functionalized poly (ionic liquid) as the support to construct a ratiometric electrochemical biosensor for the selective determination of copper ions in AD rats. Biosens. Bioelectron..

[17] Chai X., Zhang L., Tian Y. (2014). Ratiometric electrochemical sensor for selective monitoring of cadmium ions using biomolecular recognition. Anal. Chem..

[18] Qian J., Wang K., Wang C., Ren C., Liu Q., Hao N., Wang K. (2017). Ratiometric fluorescence nanosensor for selective and visual detection of cadmium ions using quencher displacement-induced fluorescence recovery of CdTe quantum dots-based hybrid probe. Sens. Actuators B Chem..

[19] Yin S., Ruffin P.B., Francis T.S. (2017). . Fiber Optic Sensors.

[20] Hegde G., Asokan S., Hegde G. (2022). Fiber Bragg grating sensors for aerospace applications: A review. ISSS J. Micro Smart Syst..

[21] Zhao H., Wang F., Han Z., Cheng P., Ding Z. (2023). Research Advances on Fiber-Optic SPR Sensors with Temperature Self-Compensation. Sensors.

[22] Cuando-Espitia N., Fuentes-Fuentes M.A., May-Arrioja D.A., Hernández-Romano I., Martínez-Manuel R., Torres-Cisneros M. (2021). Dual-point refractive index measurements using coupled seven-core fibers. J. Light. Technol..

[23] Zhu C., Zheng H., Alsalman O., Naku W., Ma L. (2023). Simultaneous and Multiplexed Measurement of Curvature and Strain Based on Optical Fiber Fabry-Perot Interferometric Sensors. Photonics.

[24] Marrujo-García S., Hernández-Romano I., Torres-Cisneros M., May-Arrioja D.A., Minkovich V.P., Monzón-Hernández D. (2020). Temperature-independent curvature sensor based on in-fiber Mach–Zehnder interferometer using hollow-core fiber. J. Light. Technol..

[25] Qi K., Zhang Y., Sun J., Wu Y. (2023). Measurements of liquid surface tension and refractive index using a tapered microfiber. Opt. Laser Technol..

[26] Guzman-Sepulveda J.R., May-Arrioja D.A., Fuentes-Fuentes M.A., Cuando-Espitia N., Torres-Cisneros M., Gonzalez-Gutierrez K., LiKamWa P. (2020). All-fiber measurement of surface tension using a two-hole fiber. Sensors.

[27] Liu Y., Feng Y., Wen J., Huang L., Dong J. (2023). Integrated fiber-optic sensor based on inscription of FBG in seven-core fiber for curvature and temperature measurements. Opt. Fiber Technol..

[28] Cuando-Espitia N., Fuentes-Fuentes M.A., Velázquez-Benítez A., Amezcua R., Hernández-Cordero J., May-Arrioja D.A. (2021). Vernier effect using in-line highly coupled multicore fibers. Sci. Rep..

[29] Park E.J., Reid K.R., Tang W., Kennedy R.T., Kopelman R. (2005). Ratiometric fiber optic sensors for the detection of inter-and intra-cellular dissolved oxygen. J. Mater. Chem..

[30] Zhao Y., Zhang H., Jin Q., Jia D., Liu T. (2022). Ratiometric Optical Fiber Dissolved Oxygen Sensor Based on Fluorescence Quenching Principle. Sensors.

[31] Steinegger A., Wolfbeis O.S., Borisov S.M. (2020). Optical sensing and imaging of pH values: Spectroscopies, materials, and applications. Chem. Rev..

[32] Rosenberg M., Laursen B.W., Frankær C.G., Sørensen T.J. (2018). A fluorescence intensity ratiometric fiber optics–based chemical sensor for monitoring pH. Adv. Mater. Technol..

[33] Zhao L., Li G., Gan J., Yang Z. (2021). Hydrogel optical fiber based ratiometric fluorescence sensor for highly sensitive pH detection. J. Light. Technol..

[34] Yuan X.L., Wu X.Y., He M., Lai J.P., Sun H. (2022). A ratiometric fiber optic sensor based on CdTe QDs functionalized with glutathione and mercaptopropionic acid for on-site monitoring of antibiotic ciprofloxacin in aquaculture water. Nanomaterials.

[35] May-Arrioja D.A., Fuentes-Fuentes M.A., Hernández-Romano I., Martínez-Manuel R., Cuando-Espitia N. (2023). Ratiometric Temperature Sensing Using Highly Coupled Seven-Core Fibers. Sensors.

[36] Van Newkirk A., Antonio-Lopez J.E., Salceda-Delgado G., Piracha M.U., Amezcua-Correa R., Schülzgen A. (2015). Multicore fiber sensors for simultaneous measurement of force and temperature. IEEE Photonics Technol. Lett..

[37] Zhou Y., Wang Y., Liu H., Chen J., Yang P., She L., Chen F., Shao J., Guan Z., Zhang Z. (2021). High-sensitive bending sensor based on a seven-core fiber. Opt. Commun..

[38] Vallejo-Carrillo R.G., Salceda-Delgado G., Torres-Torres M., Amezcua-Correa R., Antonio-Lopez J.E. (2023). Tuning of optical fiber laser based on super-mode interference in a seven-core fiber. Laser Phys..

[39] Wang X., Wang Y., Ling Q., Zhang Q., Luo W., Yu Z., Tao C., Jiang X., Chen H., Guan Z. (2023). Seven-core fiber embedded ultra-long period grating for curvature, torsion or temperature sensing. Opt. Commun..

[40] Guzman-Sepulveda J.R., May-Arrioja D.A. (2013). In-fiber directional coupler for high-sensitivity curvature measurement. Opt. Express.

[41] Salceda-Delgado G., Van Newkirk A., Antonio-Lopez J.E., Martinez-Rios A., Schülzgen A., Correa R.A. (2015). Compact fiber-optic curvature sensor based on super-mode interference in a seven-core fiber. Opt. Lett..

[42] Antonio-Lopez J.E., Eznaveh Z.S., LiKamWa P., Schülzgen A., Amezcua-Correa R. (2014). Multicore fiber sensor for high-temperature applications up to 1000 C. Opt. Lett..

[43] Van Newkirk A., Antonio-Lopez E., Salceda-Delgado G., Amezcua-Correa R., Schülzgen A. (2014). Optimization of multicore fiber for high-temperature sensing. Opt. Lett..

